# Improving the quality of health care across the health system

**DOI:** 10.2471/BLT.18.226266

**Published:** 2018-12-01

**Authors:** Shamsuzzoha B Syed, Sheila Leatherman, Nana Mensah-Abrampah, Matthew Neilson, Edward Kelley

**Affiliations:** a Department of Service Delivery & Safety, World Health Organization, avenue Appia 20, 1211 Geneva 27, Switzerland.; b Department of Health Policy and Management, University of North Carolina, Chapel Hill, United States of America.

Providing quality health services is key to achieving universal health coverage (UHC).^1^ Measuring and improving access alone is insufficient to ensure that people receive quality care^2^ and to monitor progress towards UHC.^3^

In 2018, three publications have significantly increased knowledge on the importance of the quality of health services.^4^^-^^6^ The World Health Organization (WHO), the World Bank and the Organisation for Economic Co-operation and Development (OECD), the National Academies of Sciences in the United States of America and the *Lancet Global Health* Commission all covered aspects of the quality of health systems in context of UHC and the sustainable development goals (SDGs). Authors of the reports call for quality to be a core UHC consideration, with attention to the measurement of quality at local, national and international levels. As summarized by WHO Director-General Tedros Adhanom Ghebreyesus, without quality, UHC remains an empty promise.^7^

WHO concurs with recommendations that health authorities develop a clear national direction for improving the quality of health services and establish mechanisms to measure progress. Explicit policies to address the quality of health services are needed, and where multiple quality-improvement initiatives exist, these are best combined in a systematic coordinated effort to improve care across the health system. Most national governments may need to clarify structures for governance, accountability and monitoring of effort to improve quality; to secure commitment for quality through consensus-building; and to create a culture shift in their health systems such that all providers deliver, and users demand, better quality.

To support Member States in this process, WHO has launched a global effort to promote and improve national quality policies and strategies.^8^ This initiative has published the *Handbook for national quality policy and strategy*, which has been developed with national quality directorates and technical experts and is designed to support national efforts, recognizing the varied expertise of national health authorities.^9^^,^^10^

As the form and content of specific policies will vary with each country’s context, WHO outlines a sequential approach that can be adapted to each situation. Policies on quality-improvement must be linked with existing national health priorities to help meet the most pressing demands of the population and to ensure that the quality-improvement agenda is aligned to these priorities. The definition of quality must be developed locally, through a shared understanding of challenges and ambitions. Stakeholders from across the health system need to be identified and engaged. The current state of health service quality is assessed to identify key gaps that can be strengthened. Interventions are needed across all levels of the health system, along with clarification of governance arrangements, organizational structures and the information systems necessary for measurement, performance feedback and reporting. Finally, a set of indicators have to be agreed and tracked to measure the extent to which activities are producing a higher quality of care and leading to improved health outcomes. These elements are the foundation upon which to organize national efforts to improve the quality of healthcare services, avoiding the pitfall of creating a silo around the quality-improvement agenda. WHO’s handbook emphasizes the importance of integration with existing national health policies and with relevant population- and disease-specific programmes that address quality. 

Links can be made between quality-improvement efforts and the progress towards UHC in many countries; between quality-improvement and resilient health services as a foundation for health security; between quality-improvement and service delivery in fragile, conflict-affected and vulnerable settings; and between quality-improvement and reforms of primary healthcare.^11^ The need to collect and share experience on national quality efforts and to promote innovative, context-specific solutions underpins all these endeavours. The WHO Global Learning Laboratory for Quality UHC is an important contribution to this effort.^12^

## References

[1] Resolution A/RES/67/81. Global health and foreign policy. In: Sixty seventh United Nations General Assembly, New York, 12 December 2012. New York: United Nations; 2012. Available from: http://www.un.org/en/ga/search/view_doc.asp?symbol=A/RES/67/81[cited 2108 Nov 8].

[2] Kruk ME, Kelley E, Syed SB, Tarp F, Addison T, Akachi Y. Measuring quality of health-care services: what is known and where are the gaps? Bull World Health Organ. 2017 6 1;95(6):389–389A. 10.2471/BLT.17.195099 28603302 PMC5463820

[3] Tracking universal health coverage: 2015 global monitoring report. Geneva and Washington, D.C.: World Health Organization and the World Bank; 2015. Available from: http://apps.who.int/iris/bitstream/handle/10665/174536/9789241564977_eng.pdf;jsessionid=30E00342B6001704E543582BBE6B8B3F?sequence=1 [2108 Nov 8].

[4] World Health Organization, Organisation for Economic Co-operation and Development, the World Bank. Delivering quality health services: a global imperative for universal health coverage. Geneva: World Health Organization; 2018. Available from: http://apps.who.int/iris/bitstream/handle/10665/272465/9789241513906-eng.pdf?ua=1 [cited 2108 Nov 8].

[5] Crossing the global quality chasm: improving health care worldwide. Washington, D.C.: The National Academies of Sciences, Engineering, Medicine; 2018. Available from: http://nationalacademies.org/hmd/Reports/2018/crossing-global-quality-chasm-improving-health-care-worldwide.aspx[cited 2108 Nov 8]. 30605296

[6] Kruk ME, Gage AD, Arsenault C, Jordan K, Leslie HH, Roder-DeWan S, et al. High-quality health systems in the sustainable development goals era: time for a revolution. Lancet Glob Health. 2018 11;6(11):e1196–252. 10.1016/S2214-109X(18)30386-3 30196093 PMC7734391

[7] Ghebreyesus TA. How could health care be anything other than high quality? Lancet Glob Health. 2018 11;6(11):e1140–1. 10.1016/S2214-109X(18)30394-2 30196096

[8] WHO National quality policy and strategy initiative. Geneva: World Health Organization; 2018. Available from: http://www.who.int/servicedeliverysafety/areas/qhc/nqps/en/ [2108 Nov 8].

[9] Handbook for national quality policy and strategy: a practical approach for developing policy and strategy to improve quality of care. Geneva: World Health Organization; 2018. Available from: http://apps.who.int/iris/bitstream/handle/10665/272357/9789241565561-eng.pdf?ua=1 [2108 Nov 8].

[10] National quality policy and strategy: co-defining a pathway for impact. Meeting Report. WHO Global Learning Laboratory for Quality UHC. Geneva: World Health Organization; 2017. Available from: http://apps.who.int/iris/bitstream/handle/10665/258989/WHO-HIS-SDS-2017.14-eng.pdf?sequence=1 [cited 2108 Nov 8].

[11] Quality in primary health care. Technical series on primary health care. Geneva: World Health Organization; 2018. Available from: http://www.who.int/docs/default-source/primary-health-care-conference/quality.pdf?sfvrsn=96f411e5_2 [cited 2108 Nov 8].

[12] WHO Global learning laboratory for quality UHC. Geneva: World Health Organization; 2018. Available from: http://www.who.int/servicedeliverysafety/areas/qhc/gll/en/ [cited 2108 Nov 8].

